# An optimisation of four SARS-CoV-2 qRT-PCR assays in a Kenyan laboratory to support the national COVID-19 rapid response teams

**DOI:** 10.12688/wellcomeopenres.16063.1

**Published:** 2020-07-07

**Authors:** Khadija Said Mohammed, Zaydah R. de Laurent, Donwilliams O. Omuoyo, Clement Lewa, Elijah Gicheru, Robinson Cheruiyot, Brian Bartilol, Shadrack Mutua, Jennifer Musyoki, Horace Gumba, Jedidah Mwacharo, Debra Riako, Shaban J. Mwangi, Bonface M. Gichuki, Lydia Nyamako, Angela Karani, Henry Karanja, Daisy Mugo, John N. Gitonga, Susan Njuguna, Wilson Gumbi, Brian Tawa, Metrine Tendwa, Wesley Cheruiyot, Yiakon Sein, John K. Nyambu, Shem O. Patta, Thani Suleiman Thani, Eric K. Maitha, Benson Kitole, Mohamed S. Mwakinangu, Barke S. Muslih, John Ochieng Otieno, Joyce U. Nyiro, Patience Kiyuka, Leonard Ndwiga, Kevin Wamae, Domtila Kimani, Johnstone Makale, John Mwita Morobe, Victor Osoti, Arnold W. Lambisia, Calleb Odundo, Salim Mwarumba, Martin Mutunga, Philip Bejon, Benjamin Tsofa, Charles N. Agoti, Lynette Isabella Ochola-Oyier

**Affiliations:** 1 KEMRI-Wellcome Trust Research Programme, Kilifi, 230, Kenya; 2 Department Of Health Services, Taita-Taveta County Government, Taita-Taveta, Kenya; 3 Department Of Health Services, Mombasa County Government, Mombasa, Kenya; 4 Kilifi County Hospital, Kilifi, Kenya; 5 Department Of Health Services, Kwale County Government, Kwale, Kenya; 6 Hola Referral Hospital, Tana River, Kenya; 7 King Fahd Lamu County Referral Hospital, Lamu, Kenya; 8 Nuffield Department of Medicine, Centre for Clinical Vaccinology and Tropical Medicine, Churchill Hospital, University of Oxford, Oxford, UK

**Keywords:** COVID-19, SARS-CoV-2, coronavirus, RT-PCR, diagnosis, optimisation

## Abstract

**Background:** The global COVID-19 outbreak relies on a quantitative real-time polymerase chain reaction (qRT-PCR) for the detection of severe acute respiratory syndrome coronavirus (SARS-CoV-2), to facilitate the roll-out of patient care and infection control measures. There are several qRT-PCR assays with little evidence on their comparability. We report alterations to the developers’ recommendations to sustain the testing capability in our setting, where the supply of testing reagents is limited.

**Methods:** Standards generated from a serially-diluted positive control and previously identified positive/negative samples were used to determine the optimal volumes of the qRT-PCR reagents and to evaluate the validity and performance of four assays: Charité Berlin and European Virus Archive – GLOBAL (EVAg) primer-probe sets, and DAAN and Beijing Genomics Institute (BGI) premixed commercial kits. A multiplex and singleplex RT-PCR kit was used with the two primer-probe sets and the recommended assay volumes of the two premixed kits were altered.

**Results:** In comparison to the multiplex RT-PCR kit, the singleplex RT-PCR kit combined with the primer-probe sets yielded consistent cycle threshold (Ct) values across the different titrations tested. The DAAN premixed kit produced comparable Ct values across the titrations, while the BGI kit showed incomparable Ct values and inconsistent results between batches using the manufacturer’s recommended volumes.

**Conclusion:** We achieved a 2.5-fold and 4-fold increase in the number of tests/kit for the premixed kits and the primer-probe sets, respectively. The primer-probe set assays were reliable and consistent, and we preferred a combination of an EVAg and a Berlin target. Any inconclusive result was repeated by different individuals following the same protocol. DAAN was a consistent and reliable assay even at lower concentrations from the stated recommendations. BGI in contrast, required dilution to improve its performance and was hence an assay that was used in combination with EVAg or Berlin targets.

## Introduction

The coronavirus disease 2019 (COVID-19) pandemic that began in China ^1^ is caused by a novel coronavirus, named severe acute respiratory syndrome coronavirus 2 (SARS-CoV-2) ^2^. It is an important public health concern due to its global spread and unexpected high mortality (of 411,680 globally as at 10 ^th^ June 2020) [ https://coronavirus.jhu.edu/map.html], which is compounded by the unavailability of a treatment or vaccine to control or prevent the disease. SARS-CoV-2 belongs to a wider group of coronaviruses that causes respiratory distress in animals, birds and humans ^3^. The genomic characterization of SARS-CoV-2 has shown that it is distinct from severe acute respiratory syndrome coronavirus (SARS-CoV) and the Middle East respiratory syndrome (MERS)-CoV ^4^. COVID-19 mainly affects the lower respiratory tract, which can result in fatal pneumonia ^5^. As at 10 ^th^ June 2020, there were over 7.25 million accumulated cases globally ^6^ and Africa accounted for 203,899 cases and 5,530 deaths. Of these, Kenya had reported 3094 cases and 89 fatalities ^7^ and these cases may be largely underestimated due to the limited capacity for testing ^8^.

Highly sensitive and specific diagnostics for COVID-19 can inform efforts geared towards case detection, isolation, quarantine, contact tracing and subsequent infection control measures. Many antibody and antigen detection tests are still under development and validation ^4^. Furthermore, antibody tests provide evidence of exposure to infection and do not clearly diagnose the presence of active infections for decisions to be made on treatment and isolation. Due to these limitations, quantitative reverse transcription-PCR (qRT-PCR) remains a valuable laboratory diagnostic test for COVID-19. Progress in developing specific primers and standardized laboratory protocols for COVID-19 was made possible by the availability of SARS-CoV-2 genomes early in the epidemic ^4, 9, 10^. The first qRT-PCR assay (Charité, Berlin) was subsequently developed, targeting three regions in the SARS-CoV-2 genome, including envelope (E), nucleocapsid (N) and RNA-dependent RNA polymerase (RdRp) ^11^. Other test kits in the market include the European Virus Archive – GLOBAL (EVAg) primer-probe set that targets the E and RdRp regions ^12, 13^, the DAAN kit that targets the ORF1ab and N coding regions ^14^, and the BGI kit targets the ORF1ab region ^15^.

The Kenya Medical Research Institute-Wellcome Trust Research Programme (KWTRP), Kilifi, laboratory was assigned the responsibility of providing diagnostic testing support for all Coastal counties since the outbreak started in Kenya. Currently, like many low and middle-income countries, Kenya depends on international purchases and donations of testing kits. The main limitation of this process is the delays in receiving reagents from the international manufacturers due to the global travel restrictions, resulting in an inconsistent supply of testing reagents. To mitigate these challenges, the aforementioned assays were optimised and validated to primarily distinguish between false positives and true positives. This article details the lessons learnt from using these assays and presents the optimal parameters to maximise the use of the limited kits and reagents available while still maintaining assay performance.

## Methods

### Sample collection and processing

Both nasopharyngeal (NP) (FLOQswabs, COPAN Diagnostics) and oropharyngeal (OP) swabs (COPAN Diagnostics) were collected from patients presenting to health facilities with suspected COVID-19 symptoms; contacts of positive cases in quarantine facilities and positive cases in isolation centres being monitored by the rapid response teams across the six counties. Their NP/OP swabs were placed into a single tube of 3ml Universal Transport Media (COPAN Diagnostics), initially supplied by KWTRP, and later in 2ml viral transport media, supplied by KEMRI Headquarters (Nairobi). The samples were processed in a Biosafety level 2 cabinet ^16^ at KWTRP, as per WHO guidelines.

### RNA extraction

SARS-CoV-2 RNA was extracted from the NP/OP patient samples, a positive control from a heat-inactivated culture of SARS-CoV-2 isolates from Aix-Marseille University, Marseille, France and a non-template control (nuclease-free water), using the manual QIAamp Viral RNA Mini Kit (Qiagen), and later the automated RNAeasy Mini QIAcube Kit (Qiagen) to accommodate the increase in the number of samples being tested. Extraction was done as per the manufacturers' instructions. The SARS-CoV-2 positive control RNA sample, derived from the culture, was serially diluted 10-fold to generate standards that were used in the four qRT-PCR assays for optimization and a comparison of their performance.

### Real-time PCR assays

Our first tests were conducted with the Berlin Charité Laboratory protocol ^11^, which targets the RNA dependent RNA polymerase (RdRp), envelope (E) and nucleocapsid (N) regions (Berlin protocol). Within a week of using the Berlin protocol, KWTRP had received the European Virus Archive - GLOBAL (EVAg) Kit ^12, 13^ targeting the E region. After 45 tests runs, we depleted the EVAg E primers and thereafter received the DAAN kit (DAAN Gene Co., Ltd of Sun Yat-sen University) that targeted the open reading frame (ORF)1ab and N coding regions, and also contained an internal human control gene. Following the rapid depletion of the DAAN kit due to a scale-up of testing in the coastal Counties, we received the BGI Kit (BGI Genomics Co. Ltd) that targets the ORF1ab region and contains a human housekeeping β-actin gene as an internal control. Both the BGI and DAAN kits were donations to Africa by the Jack Ma foundation through the Africa CDC to Ministries of Health in the region, whereas the EVAg and Charité Berlin primer-probe sets were procured by KWTRP.

The SARS-CoV-2 RNA qRT-PCR amplification assays were optimised using the QuantiFast Multiplex RT-PCR +R kit (Qiagen) and primer-probe sets from Charité Berlin and EVAg, as per manufacturer instructions. Given the large number of samples being tested and the long runtime required for the multiplex kit protocols (approximately 2 hours), we tested the singleplex TaqMan ^®^ Fast Virus 1-step Master Mix kit (Thermo Fisher), with an approximately 1 hour runtime, to ascertain its validity and adaptability for both the Charité Berlin and EVAg assays. We used the same primer and probe concentrations with the singleplex TaqMan ^®^ Fast Virus Master Mix kit. Both the multiplex and singleplex RT-PCR kits were available in the laboratory since they are routinely used for other viral research work at KWTRP. The commercial premixed fluorescent RT-PCR reagents from the BGI and DAAN kits were used according to the manufacturer’s instructions.

### Optimisation of the qRT-PCR assays

For optimisation of the qRT-PCR assays, we used two positive RNA samples, two negative RNA samples, a non-template control and five dilutions of the positive control RNA standards. However, for the commercial premixed kits, we included the positive and negative controls accompanying the BGI and DAAN kits. Each sample was run in duplicate. To determine the optimal concentrations and volumes of primers and probes, while maximising reagent utilization, we varied the primer and probe volumes across the Charité Berlin ( Table 1– Table 3) and EVAg ( Table 4) assays. Of note, we initially received EVAg E as primers and probes separately; later on in the testing process we received EVAg E and RdRp as a primer and probe mixed together ( Table 5). The total reaction volume was reduced from 25µl to 10µl, necessitating a subsequent reduction in the PCR buffer for the TaqMan ^®^ Fast Virus 1-step Master Mix, which contains the reverse transcription enzyme and ROX dye (probe).

**Table 1.** Charité Berlin E assay reagents.

Component	Volume (μl)		
	Neat *	0.75	0.5
Nuclease-free water	4.5	4.75	5
4x TaqMan ^®^ Fast Virus 1-Step Master Mix	2.5 *	2.5 *	2.5 *
E Sarbeco forward primer	0.4	0.3	0.2
E Sarbeco reverse primer	0.4	0.3	0.2
E Sarbeco probe	0.2	0.15	0.1
RNA template	2	2	2
Total reaction volume	10	10	10

* Half of recommended assay volumes

**Table 2.** Charité Berlin N assay reagents.

Component	Volume (μl)		
	Neat *	0.75	0.5
Nuclease-free water	3.9	4.3	4.7
4x TaqMan ^®^ Fast Virus 1-Step Master Mix	2.5	2.5	2.5 *
N Sarbeco forward primer	0.6	0.45	0.3
N Sarbeco reverse primer	0.8	0.6	0.4
N Sarbeco probe	0.2	0.15	0.1
RNA template	2	2	2
Total reaction volume	10	10	10

* Half of recommended assay volumes

**Table 3.** Charité Berlin RdRp assay reagents.

Component	Volume (µl)		
	Neat *	0.75	0.5
Nuclease-free water	3.9	4.3	4.7
4x TaqMan ^®^ Fast Virus 1-Step Master Mix	2.5	2.5	2.5 *
N Sarbeco forward primer	0.6	0.45	0.3
N Sarbeco reverse primer	0.8	0.6	0.4
N Sarbeco probe	0.2	0.15	0.1
RNA template	2	2	2
Total reaction volume	10	10	10

* Half of recommended assay volumes

**Table 4.** European Virus Archive – GLOBAL (EVAg) E gene (primers-probe set) assay reagents.

Component	Volume (µl)
	Neat
Nuclease-free water	4.3
4x TaqMan ^®^ Fast Virus 1-Step Master Mix	2.5 *
EVA-g E Forward primer	0.5
EVA-g E Reverse primer	0.5
EVA-g E probe	0.2
RNA template	2
Total reaction volume	10

* Half of recommended assay volume where 4X TaqMan ^®^ Fast Virus 1-Step Master Mix (recommended 5 µl)

**Table 5.** European Virus Archive – GLOBAL (EVAg) E and RdRP gene assay reagents with readily provided mix of forward and reverse primers and probes.

Component	Volume (µl)	
	0.75	0.5
Nuclease-free water	2.9	3.75
4x TaqMan ^®^ Fast Virus 1-Step Master Mix	2.5 *	2.5 *
Primer-probe mix	2.6	1.75
RNA template	2	2
Total reaction volume	10	10

* Half of recommended assay volume where 4X TaqMan ^®^ Fast Virus 1-Step Master Mix (recommended 5 μl)

The commercial BGI and DAAN kits have primers and probes pre-mixed in PCR reaction buffers that were supplied in limited amounts. We titrated the reaction buffer to determine the optimal concentration and volumes required for the detection of SARS-CoV-2 RNA from clinical samples. The recommended volume of the enzyme mix (Hot Start Taq DNA polymerase and c-MMLV reverse transcriptase) per reaction for the DAAN kit was 3µl. We reduced this to 0.5µl and consistently used this alongside varying volumes of the PCR reaction mix (Liquid A) containing the primers and probes ( Table 6). For the BGI kit, in addition to titrating the PCR reaction mix ( Table 7), we went further to assess its validity by also altering the amount of enzyme mix, testing two conditions, 0.5µl and 0.25µl, of the enzyme mix while maintaining the PCR reaction mix volume, as shown in Table 8. For all the kits, we tested three titration points across the four RT-PCR assays as follows: neat (50% of the manufacturer’s recommendations), 75% of the neat and 50% of the neat.

**Table 6.** DAAN assay reagents.

Component	Volume (µl)		
	Neat*	0.75	0.5
Nuclease-free water	**-**	1.1	3.2
Reaction mix (Liquid A)	8.5	6.4	4.3
Enzyme mix (Liquid B)	0.5	0.5	0.5
RNA template	2	2	2
Total reaction volume	11	10	10

* Half of recommended assay volume of the reaction mix (recommended volume is 17 μl)

**Table 7.** BGI assay reagents.

Component	Volume (μl)			Altered enzyme volume (μl)	
	Neat *	0.75	0.5	Mix 1	Mix 2
Reaction mix	9.3	7	4.7	0.5	0.75
Enzyme mix	0.8	0.8	0.8	0.25	0.25
RNA template	2	2	2	2	2
Reaction mix				7	7
Total reaction volume	12.1	9.8	7.5	10	10

* Half of recommended assay volume of the reaction mix (recommended volume is 18.5 μl)

**Table 8.** BGI assay reagents with altered enzyme mix volumes.

Component	Volume (µl)	
	Mix1	Mix2
Nuclease free water	0.5	0.75
Reaction mix	7	7
Enzyme mix	0.5	0.25
RNA template	2	2
Total reaction volume	10	10

All these assays were run on the Applied Biosystems™ 7500 Real-Time PCR System and analysed on the 7500 software v2.3. The qRT-PCR conditions are indicated in Table 9. The annealing and extension conditions varied depending on the gene targets and detection assays. For the analysis of the amplification plots and subsequent data, different baseline points and thresholds were set manually as illustrated in Table 10.

**Table 9.** Quantitative reverse transcription-PCR (qRT-PCR) cycling conditions for detection of SARS-CoV-2 RNA using four assays.

Step	Berlin Charité (E, N and RdRP)		EVAg (E and RdRp)		BGI (ORF1ab)	DAAN (ORF1ab and N)
	QuantiFast RT-PCR +R	TaqMan ^®^ Fast Virus	QuantiFast RT-PCR +R	TaqMan ^®^ Fast Virus	Kit component	Kit component
Reverse transcription	55°C	50°C	55°C	50°C	50°C	50°C
	20 min	5 min	20 min	5 min	20 mins	15 mins
Activation	95°C	95°C	95°C	95°C	95°C	95°C
	5 min	20 sec	5 min	20 sec	10 mins	15 mins
Denaturation	95°C	95°C	95°C 15 sec 45 cycles	95°C	95°C	94°C
	15 sec	3 sec		3 sec	15 sec	15 sec
	45 cycles	40 cycles		40 cycles	40 cycles	45 cycles
Annealing and extension	58°C	60°C	58°C	58°C	60°C	55°C
	30 sec	30 sec	45 sec	45 sec	30 sec	45 sec
	45 cycles	40 cycles	40 cycles	40 cycles	40 cycles	45 cycles

**Table 10.** ABI 7500 Real-Time PCR System analysis settings for detection of SARS-CoV-2 RNA using four assays. Charité Berlin primer-probe sets (E, N and RdRp genes), European Virus Archive – GLOBAL (EVAg) primer-probe sets (E and RdRp genes), DAAN kit (ORF1ab region and N gene) and BGI kit (ORF1ab region) based on the standard curves.

	TaqMan® Fast Virus 1-step Master Mix RT-PCR Kit					QuantiFast Multiplex RT-PCR +R Kit		DAAN RT-PCR Kit	BGI RT-PCR Kit
	Berlin E	Berlin N	Berlin RdRp	EVAg E	EVAg RdRp	Berlin RdRp	EVAg E		
Baseline starting point	3	3	3	3	3	3	3	3	3
Baseline ending point	20	24	19	18	19	12	12	22	18
Threshold	0.54	0.02	0.02	0.58	0.09	0.21	2.47	16271	110241
Positive sample cut-off Ct value	35	36	31	37	36	35	38	N gene – 39 ORF1ab - 40	34

### Ethical statement

Ethical approval was not required since the patient samples used in the optimisation assays were part of the testing process for ensuring consistency, replicability and the validity of all the testing kits used. These optimisations allowed us to develop testing SOPs to ensure we report a valid result to the COVID-19 county rapid response teams.

## Results

### Primer-probe set assays

**Figure 1.**
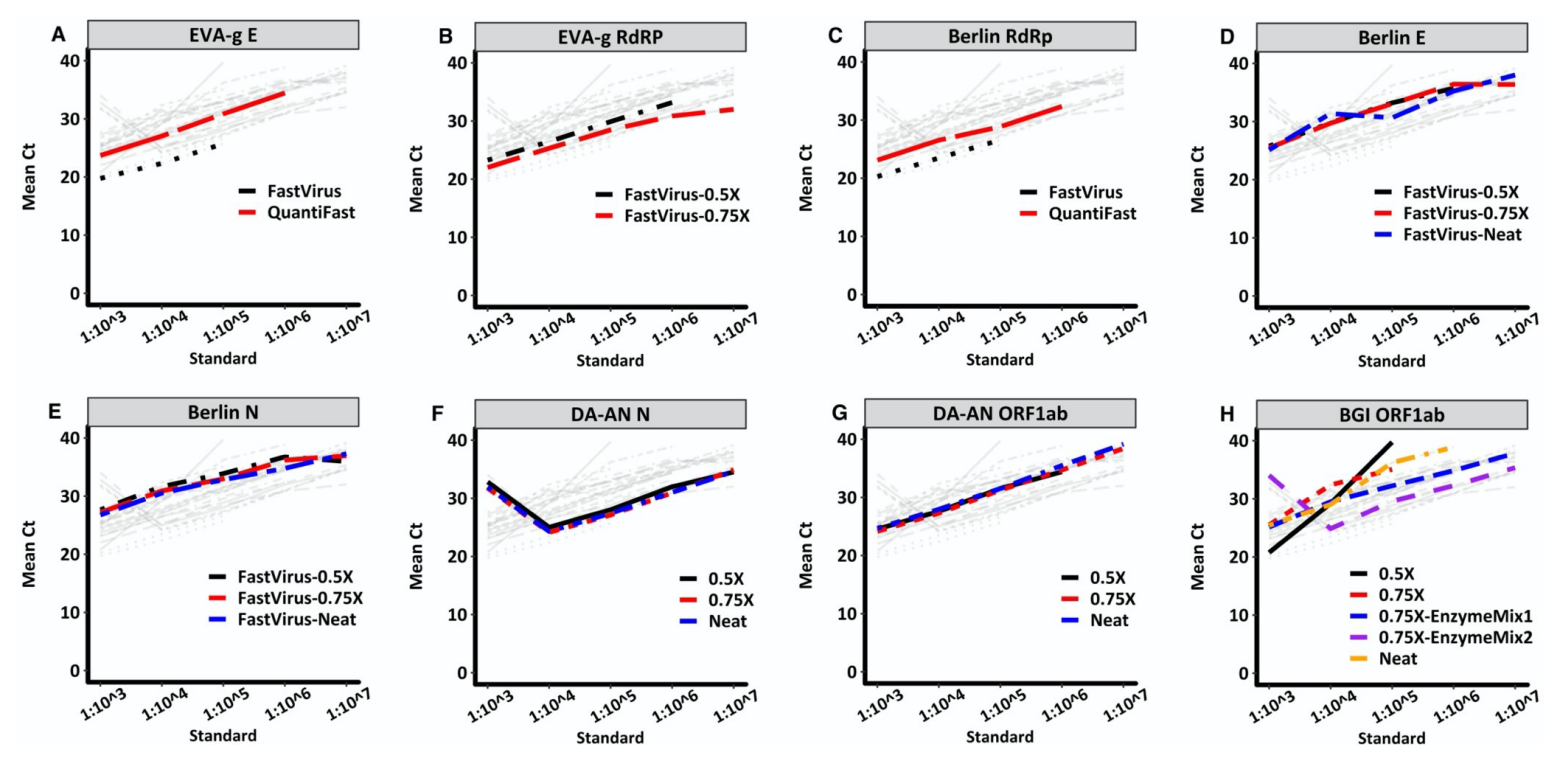
Standard curves of 10-fold dilutions and their corresponding Ct values resulting from titrating RT-PCR reagents from four assays. Charité Berlin assay targeting E gene ( **D**), N gene ( **E**) and RdRp gene ( **A**); EVA-g, targeting E gene ( **B**) and RdRp gene ( **C**); DAAN, targeting N gene ( **F**) and ORF1ab region ( **G**) and BGI, targeting ORF1ab region ( **H**). All assays indicated “Neat” used half the concentration of the recommended assay volume and those indicated “0.5X” or “0.75X” used 0.5 times and 0.75 times the volume of the “Neat” assay volume, respectively. The primer-probe set assays are used alongside commercial RT-PCR kits TaqMan® Fast Virus 1-Step Master Mix and QuantiFast Multiplex RT-PCR +R Kit. In the BGI assays (panel H), “EnzymeMix1” and “EnzymeMix2” assays were similar to the “0.75X” assay, however, the enzyme mix volumes used were 0.5ul and 0.25ul, respectively. The light grey lines in each plot illustrate comparisons of all other assays versus the respective coloured plot assay.

**Figure 2.**
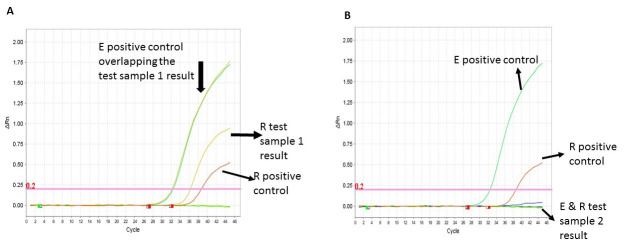
Amplification curves of the two target gene approach. The EVAg E and Berlin RdRp assays were run under the same RT-PCR conditions in the same PCR 96-well plate. ( **A**) Shows a definitive positive result for test sample 1 for the E target region (orange line, Ct 32.2) underlying the green line (the E positive control, Ct 32.4) and the R region (yellow line, Ct 36.3) and R positive control (red line, Ct 38.7). ( **B**) Shows a definite negative result for test sample 2 with no amplification curves when compared to the E positive control (green line) and the R positive control (red line).

There were differences between the multiplex (QuantiFast RT-PCR +R Kit) and singleplex (TaqMan® Fast Virus 1-Step Master Mix Kit) kits for both the Charité Berlin and EVAg primer-probes sets. Compared with the singleplex kit assay ( *Underlying data:* Data file 1 ^17^), the multiplex EVAg E gene assay showed delayed Ct values ( *Underlying data:* Data file 2 ^17^) potentially due to differences in the assays’ chemistry, the differential salt concentration and pH. The singleplex kit, therefore, appeared to have improved sensitivity compared to the multiplex kit ( Figure 1A). Using the singleplex kit and maintaining the same PCR conditions, we titrated the primer-probe volumes to determine the best concentration with high signal to noise ratio. We used primer concentrations in the 200nM-600nM range, and probe concentrations between 75nM-200nM ( *Underlying data:* Data file 3 ^17^). The EVAg RdRp primer-probe set showed similar Ct values across the titrations tested and 50% of the neat recommendations were used to extend the number of tests conducted with this assay ( *Underlying data:* Data file 4 ^17^; Figure 1B). Notably, the Berlin RdRP target assay inconsistently detected positive sample replicates and did not report cycle threshold (Ct) values for known positive samples in the multiplex assay ( *Underlying data:* Data file 5 ^17^). However, the singleplex assay was able to detect only one positive sample and all the standards ( *Underlying data:* Data file 6 ^17^), while generally reporting earlier Ct values ( Figure 1C). The Berlin E assay had comparable mean Ct values across the titrations with consistent performance ( *Underlying data:* Data file 7 ^17^; Figure 1D). On the other hand, the Berlin N assay resulted in mean Ct values with a difference of 1 in some samples and standards across the titrations ( *Underlying data:* Data file 8 ^17^; Figure 1E). Consequently, we settled for the lowest titration point (50% of neat volume) for the Berlin E assay and 75% of the neat volume for the Berlin N assay as our optimal volume for subsequent testing to get the maximum number of assays for the reagents available. We also determined that running two target regions concurrently saved on time and allowed us to definitively and rapidly identify a positive result. This was evident with the EVAg E and RdRp assays, and this combination increased our confidence in calling a positive test result ( Figures 2A and 2B).

**Figure 3.**
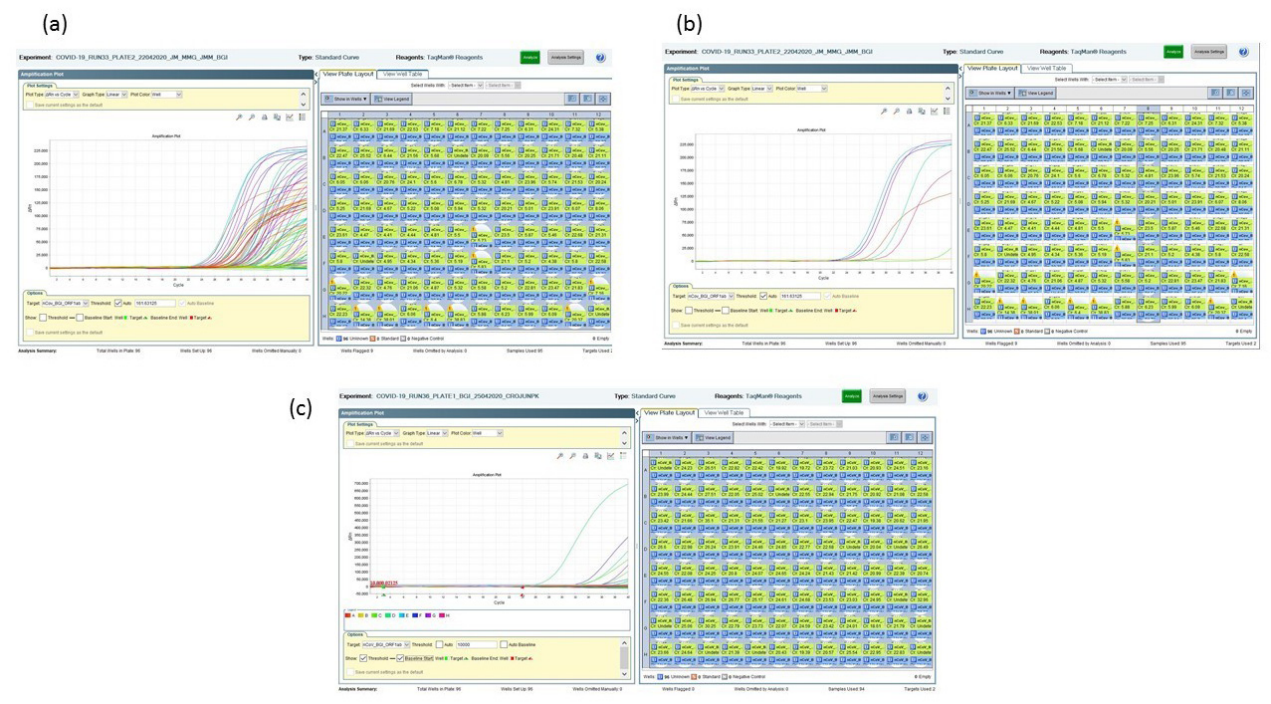
BGI assay amplification plots using 50% of the manufacturer’s recommendation. ( **A**) The multicoloured curves indicate a large number of positive results from a 96 sample test run. ( **B**) A zoom in on a single column of 8 test samples indicates at least 6 test samples as being SARS-CoV-2 positive. ( **C**) Following the use of 75% of neat volumes and 0.25µl of the enzyme mix showed improved sensitivity in the assay as indicated from the 96 sample test run with. Only 4 samples testing positive using a Ct cut-off of <34.

### Commercial premixed test kits

For the BGI and DAAN premixed kits, the quantities supplied conducted about 50 and 96 tests per kit, respectively. The kits were used at 50% of the manufacturer’s recommendation to maximise the number of tests per kit. Upon further optimization, it was clear that half, quarter and even an eighth of the manufacturer’s recommended volumes produced similar results for the DAAN kit ( *Underlying data:* Data files 9 and 10 ^17^; Figures 1F and 1G). Since the DAAN kit is a three-gene target assay, it proved to be an efficient assay in confirming the presence of SARS-CoV-2 RNA and the quality of the sample taken using the presence of the human gene internal control.

We noted batch to batch variation with the BGI kit when we used 50% of the manufacturer’s recommended volumes. As shown in Figures 3A and 3B, a large number of the samples tested (>70% of the samples) were positive, and we considered this to be unlikely, and thus ran confirmatory testing. The confirmatory second test with either Berlin E or Berlin N primer-probe set assay did not yield the equivalent number of positives as the BGI test, and we therefore concluded the initial results were false positives. The titration of the assay downwards to 75% of the neat reaction volumes and scaling down the enzyme mix by a third improved the specificity of the test ( Figure 3C), as there was a reduction in the number of false positives. Further titrations down to 25% of the manufacturer’s recommendations (50% of neat) yielded a difference of >2 Ct values between this titration and the neat and 75% titrations for the standards, while the previously identified positive samples and internal control were not detected ( *Underlying data:* Data file 11 ^17^). The results across different enzyme mix titrations were consistent in their detection of the samples and internal controls in several runs as well as the serially-diluted positive ( *Underlying data:* Data file 12 ^17^; Figure 1H).

Based on the 10-fold serial dilutions of the positive control SARS-CoV-2 RNA, we established assay-specific Ct value cut-offs ( Table 10) to determine a positive result, since the assays have different levels of signal-to-noise ratio.

## Discussion

Our experience from over 15,500 tests has allowed us to develop a series of adjustments to the assays to optimize their use in testing for SARS-CoV-2. We settled on a two-target gene assay to improve the detection of SARS-CoV-2 RNA. This finding was similar to Corman *et al*. (2020), where they suggested that the E region could be used as an initial screening tool, followed by a confirmatory test with the RdRp assay ^11^. Our ranking of the individual test performance based on calling a true positive was as follows: EVAg E, Berlin E, EVAg RdRp, Berlin N and Berlin RdRp. However, we settled on a combination of two targets being positive for a definitive positive test result. If one of the targets was negative it was considered an inconclusive result and was repeated by an independent team with a different assay. As the scale of testing increased from receiving tens of samples a day to more than 400 per day, our test ranking allowed us to settle on a single test for a quicker turn-around-time of validated results and at the same time maximising resources. For the DAAN (premixed) assay we used the same workflow since it was a combined two targets assay and any inconclusive results were repeated with a different test, EVAg or Berlin assays. In addition, this kit had the advantage of the human internal control gene to further ensure the validity of the test result. 

Even though the BGI kit sensitivity was improved when run according to the dilutions that we described above, rather than the manufacturer’s instructions, the assay still had a low signal-to-noise ratio at the high Ct value range, making it difficult to confidently call positives. We determined that a positive result was more reliable when Ct values were lower than 34, but unreliable with higher Ct values. We therefore always combined BGI with a more reliable assay, EVAg RdRp, Berlin N or Berlin RdRp (in this order depending on which primers were available), since at the time of receiving this assay we had depleted EVAg E and DAAN and were low on the E Berlin test kit and needed to economize their use.

For quality control during testing, we included in every assay a negative template control, two positive extraction controls (a neat and 1 in 10 dilution) and 2 negative controls placed randomly (but not near each other) across the wells of a 96-well format plate. Furthermore, any repeat assays were conducted by an independent team, emphasizing the importance of well-trained personnel that are required to conduct the testing and to ensure the thorough and careful set up of the assay to minimise contamination. Since it is a two-step procedure (RNA extraction and qPCR) any errors will have downstream consequences during the labour-intensive testing process. To create a seamless pipeline for COVID-19 testing, sample and data management teams were required to collate the qRT-PCR test result with the patient data and produce an individual result report for each sample tested.

## Conclusions

We achieved a 2.5-fold and 4-fold increase in the number of tests per kit for the commercial premixed kits and primer-probe sets, respectively, by adjusting the manufacturer’s recommendations on quantities following careful optimization in our laboratory. This enabled us to continuously conduct and support testing in the Coastal region of Kenya and address the challenge of inconsistencies in the supply of testing reagents. We highlight the challenges encountered in the use of the BGI kit that we noted was prone to false positives, but this was mitigated by diluting the reagent volumes and by including an additional confirmatory assay. Due to the nature of the qPCR assay, any kit may lead to false positives and thus in addition to negative controls, a dilution series of the positive controls, a confirmatory test and a set threshold must all be included to more confidently report a positive test result. Assays should be repeated where the Ct value falls into the indeterminate range. A key lesson learnt, when rapidly setting up and scaling up novel testing kits in a pandemic, is the need for sufficient, well-trained and dedicated staff to optimise and conduct the test and the requirement of a specialist data management team.

## Data availability statement

### Underlying data

Havard Dataverse: An optimisation of four SARS-CoV-2 qRT-PCR assays in a Kenyan laboratory to support the national COVID-19 rapid response teams, https://doi.org/10.7910/DVN/WPZHQR ^17^.

This project contains the following underlying data:

- Data_File_1 -_EVA-g_E_(Fast Virus 1)
- Data_File_2 -_EVA-g_E_(QuantiFast)
- Data_File_3 -_EVA-g_E_(Fast Virus 2)
- Data_File_4 -_EVA-g_RdRp_(FastVirus)
- Data_File_5 -_Berlin_RdRp_(QuantiFast)
- Data_File_6 -_Berlin_RdRp_(Fast_Virus)
- Data_File_7 -_Berlin_E_(FastVirus)
- Data_File_8 -_Berlin_N_(FastVirus)
- Data_File_9 -_DAAN_N
- Data_File_10 -_DAAN_ORF1ab
- Data_File_11 -_BGI1-ORF1ab
- Data_File_12 -_BGI2-ORF1ab

Data are available under the terms of the Creative Commons Zero "No rights reserved" data waiver (CC0 1.0 Public domain dedication).
