# An optimization of four SARS-CoV-2 qRT-PCR assays in a Kenyan laboratory to support the national COVID-19 rapid response teams

**DOI:** 10.12688/wellcomeopenres.16063.2

**Published:** 2022-03-04

**Authors:** Khadija Said Mohammed, Zaydah R. de Laurent, Donwilliams O. Omuoyo, Clement Lewa, Elijah Gicheru, Robinson Cheruiyot, Brian Bartilol, Shadrack Mutua, Jennifer Musyoki, Horace Gumba, Jedidah Mwacharo, Debra Riako, Shaban J. Mwangi, Bonface M. Gichuki, Lydia Nyamako, Angela Karani, Henry Karanja, Daisy Mugo, John N. Gitonga, Susan Njuguna, Wilson Gumbi, Brian Tawa, Metrine Tendwa, Wesley Cheruiyot, Yiakon Sein, John K. Nyambu, Shem O. Patta, Thani Suleiman Thani, Eric K. Maitha, Benson Kitole, Mohamed S. Mwakinangu, Barke S. Muslih, John Ochieng Otieno, Joyce U. Nyiro, Patience Kiyuka, Leonard Ndwiga, Kevin Wamae, Domtila Kimani, Johnstone Makale, John Mwita Morobe, Victor Osoti, Arnold W. Lambisia, Calleb Odundo, Salim Mwarumba, Martin Mutunga, Philip Bejon, Benjamin Tsofa, Charles N. Agoti, Lynette Isabella Ochola-Oyier

**Affiliations:** 1 KEMRI-Wellcome Trust Research Programme, Kilifi, Kenya; 2 Department Of Health Services, Taita-Taveta County Government, Taita-Taveta, Kenya; 3 Department Of Health Services, Mombasa County Government, Mombasa, Kenya; 4 Kilifi County Hospital, Kilifi, Kenya; 5 Department Of Health Services, Kwale County Government, Kwale, Kenya; 6 Hola Referral Hospital, Tana River, Kenya; 7 King Fahd Lamu County Referral Hospital, Lamu, Kenya; 8 Nuffield Department of Medicine, Centre for Clinical Vaccinology and Tropical Medicine, Churchill Hospital, University of Oxford, Oxford, UK

**Keywords:** COVID-19, SARS-CoV-2, coronavirus, qRT-PCR, diagnosis, optimization

## Abstract

**Background:** The COVID-19 pandemic relies on real-time polymerase chain reaction (qRT-PCR) for the detection of Severe Acute Respiratory Syndrome Coronavirus 2 (SARS-CoV-2), to facilitate roll-out of patient care and infection control measures. There are several qRT-PCR assays with little evidence on their comparability. We report alterations to the developers’ recommendations to sustain the testing capability in a resource-limited setting.

**Methods:** We used a SARS-CoV-2 positive control RNA sample to generate several 10-fold dilution series that were used for optimization and comparison of the performance of the four qRT-PCR assays: i) Charité Berlin primer-probe set, ii) European Virus Archive – GLOBAL (EVAg) primer-probe set, iii) DAAN premixed commercial kit and iv) Beijing Genomics Institute (BGI) premixed commercial kit. We adjusted the manufacturer- and protocol-recommended reaction component volumes for these assays and assessed the impact on cycle threshold (Ct) values.

**Results:** The Berlin and EVAg E gene and RdRp assays reported mean Ct values within range of each other across the different titrations and with less than 5% difference. The DAAN premixed kit produced comparable Ct values across the titrations, while the BGI kit improved in performance following a reduction of the reaction components.

**Conclusion:** We achieved a 2.6-fold and 4-fold increase in the number of tests per kit for the commercial kits and the primer-probe sets, respectively. All the assays had optimal performance when the primers and probes were used at 0.375X, except for the Berlin N gene assay. The DAAN kit was a reliable assay for primary screening of SARS-CoV-2 whereas the BGI kit’s performance was dependent on the volumes and concentrations of both the reaction buffer and enzyme mix. Our recommendation for SARS-CoV-2 diagnostic testing in resource-limited settings is to optimize the assays available to establish the lowest volume and suitable concentration of reagents required to produce valid results.

## Introduction

The coronavirus disease 2019 (COVID-19) pandemic that began in China ^1 ^ is caused by a novel coronavirus, named Severe Acute Respiratory Syndrome Coronavirus 2 (SARS-CoV-2) ^2^. It is an important public health concern due to its global spread and unexpected high mortality (of 411,680 globally as at 10 ^th^ June 2020) [ https://coronavirus.jhu.edu/map.html], which is compounded by the unavailability of a treatment or vaccine to control or prevent the disease at the time of writing this paper, early in the pandemic. SARS-CoV-2 belongs to a wider group of coronaviruses that causes respiratory distress in animals, birds and humans ^3^. Its genomic characterization has shown that it is distinct from severe acute respiratory syndrome coronavirus (SARS-CoV) and the Middle East respiratory syndrome (MERS) ^4^. COVID-19 mainly affects the lower respiratory tract, which can result in fatal pneumonia ^5^. By 10 ^th^ June 2020, there were over 7.25 million accumulated cases globally ^6 ^ and Africa accounted for 203,899 cases and 5,530 deaths. Of these, Kenya had reported 3094 cases and 89 fatalities ^7^. The number of cases may be largely underestimated due to the limited capacity for testing ^8^.

Highly sensitive and specific diagnostics for COVID-19 can inform efforts geared towards case detection, isolation, quarantine, contact tracing and subsequent infection control measures. Many antibody and antigen detection tests are still under validation at this time ^4^. Furthermore, antibody tests provide evidence of exposure to infection and do not clearly diagnose the presence of active infections for decisions to be made on treatment and isolation. Due to these limitations, quantitative reverse transcription-PCR (qRT-PCR) remains a valuable laboratory diagnostic test for COVID-19. Progress in developing specific primers and standardized laboratory protocols for COVID-19 was made possible by the availability of SARS-CoV-2 genomes early in the epidemic ^4, 9, 10^. The first qRT-PCR assay (Charité, Berlin) was subsequently developed by the Charité Institute of Virology, Universitätsmedizin Berlin, and it targets three regions in the SARS-CoV-2 genome, including envelope (E), nucleocapsid (N) and RNA-dependent RNA polymerase (RdRp) ^11^. Subsequently, other testing kits were developed and introduced into the market: including the European Virus Archive – GLOBAL (EVAg) primer-probe set that targets the E and RdRp regions ^12, 13^, the DAAN kit (DAAN Gene Co. Ltd of Sun Yat-sen University) targets the ORF1ab and N coding regions ^14^, and the BGI kit (BGI Genomics Co. Ltd) targets the ORF1ab region ^15^.

The Kenya Medical Research Institute-Wellcome Trust Research Programme (KWTRP), Kilifi, laboratory was assigned the responsibility of providing diagnostic testing support for all Coastal counties since the outbreak started in Kenya. Currently, like many low and middle-income countries, Kenya depends on international purchases and donations for testing kits. The main limitation of this process is the delays in receiving reagents from the international manufacturers due to the global travel restrictions, resulting in an inconsistent supply of testing reagents. To mitigate these challenges, the aforementioned assays were optimized to primarily establish the lowest volume and suitable concentration of reagents required to produce valid results. This article details the lessons learnt from using these assays early in the pandemic and presents the optimal parameters to maximize the use of the limited kits and reagents available while still maintaining assay validity.

## Methods

### RNA extraction

Ribonucleic acid (RNA) was extracted from the positive control, a SARS-CoV-2 heat-inactivated culture supernatant donated by Aix-Marseille University, Marseille, France) and a non-template control (nuclease-free water) using QIAamp Viral RNA Mini Kit (Qiagen). Extraction was done as per the manufacturers' instructions. The positive control RNA sample was used to generate 10-fold dilution series that were used for optimization and comparison of the performance of the four qRT-PCR assays.

### Real-time PCR assays modifications

We adjusted the manufacturer- and protocol-recommended reaction component volumes for all the assays and assessed the impact on cycle threshold (Ct) values. The assays used included the Berlin targeting E, N and RdRp genes individually, European Virus Archive (EVAg) targeting E and RdRp genes individually, the DAAN kit targeting the ORF1ab and N coding regions simultaneously, and the BGI kit targeting the ORF1ab region. We titrated the primers and probes to achieve the three concentrations to be validated relative to the manufacturer-recommended primer and probes concentration of 1X. The three titration points are herein referred to as 0.5X. 0.375X and 0.25X. In all the assays, we carried out duplicate reactions of two positive RNA samples, two negative RNA samples, a non-template control and five 10-fold dilution series of the positive control RNA.

### Berlin and EVAg assays titrations

The original protocol employed Superscript III One Step RT-PCR system with Platinum Taq Polymerase for both assays. These reagents were not available in our lab and thus we opted for TaqMan ^®^ Fast Virus 1-step Master Mix (Applied Biosystems) in a 10µl total reaction volume (final working concentration of 1X). To determine the optimal concentrations and volumes of primers and probes, these were varied for both Berlin ( Table 1) and EVAg ( Table 2) assays while holding the TaqMan master mix, template and total reaction volumes constant. The EVAg E and RdRp assays were later supplied as a mix of forward and reverse primers and probes (primer-probe set), so these were only tested at 0.375X and 0.25X.

**Table 1.** Titrated volumes of Charité Berlin primers and probes using a standard volume of TaqMan Fast Virus 1-step RT-PCR master mix.

Component	Volume (μl)								
	E gene assay			N gene assay			RdRp gene assay		
	0.5X	0.375X	0.25X	0.5X	0.375X	0.25X	0.5X	0.375X	0.25X
4X TaqMan master mix	2.5	2.5	2.5	2.5.	2.5	2.5.	2.5	2.5	2.5
Forward primer	0.4	0.3	0.2	0.6	0.45	0.3	0.6	0.45	0.3
Reverse primer	0.4	0.3	0.2	0.8	0.6	0.4	0.8	0.6	0.4
Probe	0.2	0.15	0.1	0.2	0.15	0.1	0.2	0.15	0.1
Nuclease free water	4.5	4.75	5	3.9	4.3	4.7	3.9	4.3	4.7
RNA template	2	2	2	2	2	2	2	2	2
Total reaction volume	10	10	10	10	10	10	10	10	10

**Table 2.** Titrated volumes of EVAg primers and probes using a standard volume of TaqMan Fast Virus 1-step RT-PCR master mix.

Component	Volume (μl)		
	E gene assay	E/RdRp gene primer-probe mix	
	0.5X	0.375X	0.25X
4X TaqMan master mix	2.5	2.5	2.5
Forward primer	0.5	2.6	1.75
Reverse primer	0.5		
Probe	0.2		
Nuclease free water	4.3	2.9	3.75
RNA template	2	2	2
Total reaction volume	10	10	10

### BGI and DAAN kits titrations

The commercial BGI and DAAN kits have primers and probes provided as a premix in the PCR reaction mix and these were supplied in limited amounts. Therefore, the reaction mix (Liquid A) for DAAN was titrated to 0.5X, 0.375X and 0.25X. The recommended volume of the Liquid B (Hot Start Taq DNA polymerase and c-MMLV reverse transcriptase) per reaction for the DAAN kit was 3 µl (1X). Given the limited quantity provided, this was reduced to 0.5 µl (0.16X) across all the varying volumes of Liquid A ( Table 3). 

**Table 3.** Titration volumes of PCR reaction mix (Liquid A) and enzyme mix (Liquid B) of the DAAN kit.

Component	Volume (μl)		
	0.5X	0.375X	0.25X
Reaction mix (Liquid A)	8.5	6.4	4.3
Enzyme mix (Liquid B)	0.5	0.5	0.5
Nuclease free water	0	1.1	3.2
RNA template	2	2	2
Total reaction volume	10	10	10

Prior use of the BGI assay as per the manufacturer’s recommended protocol generated a high signal-to-noise ratio necessitating optimization. The recommended volume of the enzyme mix per reaction for this kit was 1.5µl (1X). However, we initially reduced this to 0.8µl (0.5X) and maintained this volume alongside varying volumes of the PCR reaction mix. We further tested two enzyme mix volumes of 0.5µl (0.33X) and 0.25µl (0.16X), while maintaining the reaction mix volume for the 0.375X concentration ( Table 4).

**Table 4.** Titration volumes of PCR reaction mix and enzyme mix from the BGI kit.

Component	Volume (μl)			Altered enzyme mix volume (μl) at 0.375X of the reaction mix		
	0.5X	0.375X	0.25X	Mix 1 (0.33X)	Mix 2 (0.16X)
Reaction mix	9.3	7.0	4.7	7.0	7.0
Enzyme mix	0.8	0.8	0.8	0.5	0.25
Nuclease free water	0	0	0	0.5	0.75
RNA template	2	2	2	2	2
Total reaction volume	10	10	10	10	10

### Cycling conditions

All these assays were run on the Applied Biosystems™ 7500 Real-Time PCR System and analyzed using the 7500 software v2.3. The manufacturer’s recommended qRT-PCR conditions are indicated in Table 5.

**Table 5.** Quantitative reverse transcription-PCR (qRT-PCR) cycling conditions for detection of SARS-CoV-2 RNA using four assays.

Step	Charité Berlin (E, N and RdRp)	EVAg (E and RdRp)	BGI (ORF1ab)	DAAN (ORF1ab and N)
	TaqMan ®Fast Virus 1-step master mix	TaqMan ^®^Fast Virus 1-step master mix	Kit component	Kit component
Reverse transcription	50°C	50°C	50°C	50°C
	5 min	5 min	20 mins	15 mins
Activation	95°C	95°C	95°C	95°C
	20 sec	20 sec	10 mins	15 mins
Denaturation	95°C	95°C	95°C	94°C
	3 sec	3 sec	15 sec	15 sec
	40 cycles	40 cycles	40 cycles	45 cycles
Annealing and extension	60°C	58°C	60°C	55°C
	30 sec	45 sec	30 sec	45 sec
	40 cycles	40 cycles	40 cycles	45 cycles

Based on the 10-fold serial dilutions of the positive control SARS-CoV-2 RNA, we established assay-specific Ct value cut-offs to determine a positive result, since the assays have different levels of signal-to-noise ratio. For the analysis of the amplification plots and subsequent data, different baseline points and thresholds were set manually as illustrated in Table 6.

**Table 6.** ABI 7500 Real-Time PCR System analysis settings for detection of SARS-CoV-2 RNA using four assays based on the standard curves.

Parameters	TaqMan® Fast Virus 1-step Master Mix Kit				DAAN Kit		BGI Kit
	Berlin E	Berlin N	EVAg E	EVAg RdRp	N gene	ORF1ab	ORF1ab
Baseline starting point	3	3	3	3	3	3	3
Baseline ending point	20	24	18	19	22	22	18
Threshold	0.54	0.02	0.58	0.09	16271	16271	110241
Positive sample cut-off Ct value	35	36	37	36	39	40	34

## Results

### Impact of titrations on the four assays

We assessed the effect of the assay modifications on the overall sensitivity of the results. We did not evaluate the performance of the assays using the recommended manufacturer’s volumes, since they had been proven to work during routine testing. The focus was on deriving the smallest volume of reagents required to correctly identify a positive case.

**Figure 1.**
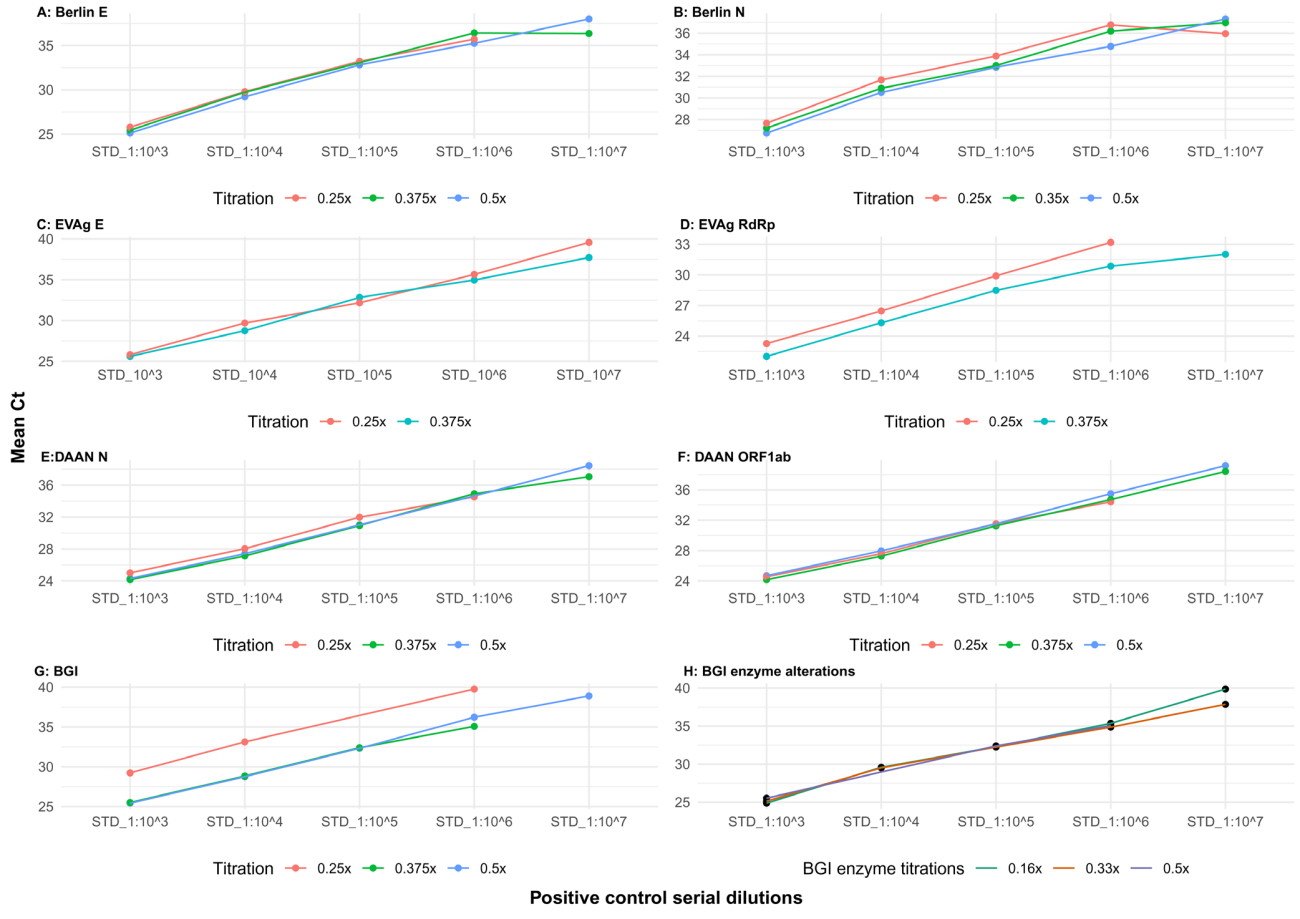
Gene-specific concordance assessment of four SARS-CoV-2 qRT-PCR assays. The plots show the comparison of qRT-PCR Ct values for the different assays across five 10-fold dilution series of the positive control RNA. The mean Ct values for duplicates tested for each sample are shown on the y-axis. 0.5X, 0.375X and 0.25X represent fractions of the recommended volumes of primer and probe.

The Berlin E and N gene assays were assessed at three titrations - 0.5X, 0.375X and 0.25X. The E gene assay had comparable mean Ct values across the titrations with consistent performance. At 0.5X, all the dilution series of the positive control RNA were detected. The 0.25X titration was not notably different from 0.375X, although the former did not detect the last positive control dilution of 1:10 ^7^ ( *Underlying data:* Data file 1 ^16^; Figure 1A). Given that 0.375X of primers and probes detected all the dilution series of the positive control in a consistent trend and with little Ct difference between replicates, we used this to set our cut-off for positivity going forward. The Berlin N gene assay was more consistent, with all the titrations detecting all dilution series of the positive control RNA ( *Underlying data:* Data file 2 ^16^; Figure 1B). However, the 0.5X titration showed a more consistent trend in amplification, with little Ct value difference between replicates and between the other titrations. Consequently, we settled for this titration volume for subsequent testing.

The EVAg E and RdRp gene (primer-probe set) assays were assessed at two titrations - 0.375X and 0.25X. For the E gene assay, both titrations detected all the dilution series of the positive control RNA. There was no notable difference between the two titrations ( *Underlying data:* Data file 3 ^16^; Figure 1C). We settled for 0.375X as our optimal volume of the primer-probe mix since all the dilution series of the positive control were detected in a consistent trend and with little Ct difference between replicates. For the RdRp gene assay, the 0.25X titration did not detect the last dilution point (1:10 ^7^) whereas, the 0.375X titration was more consistent and detected all the dilutions series ( *Underlying data:* Data file 4 ^16^; Figure 1D). Thus, the 0.375X volume was chosen for subsequent testing.

The BGI and DAAN premixed kits supplied conducted about 50 and 96 tests per kit, respectively. In the dual-gene target DAAN assay, three titrations - 0.5X, 0.375X and 0.25X were assessed. All the replicates of the dilution series of the positive control RNA had little Ct value differences (less than 1) across the titrations for both the N and ORF1ab genes ( Figure 1E and 1F). The 0.5X and 0.375X titrations detected all the positive control RNA dilution series, while the 0.25X volume failed to detect the last positive control dilution of 1:10 ^7^ for both gene targets ( *Underlying data:* Data file 5 and 6 ^16^). Consequently, we settled for 0.375X titration for subsequent runs, yielding 252 tests per kit.

**Figure 2.**
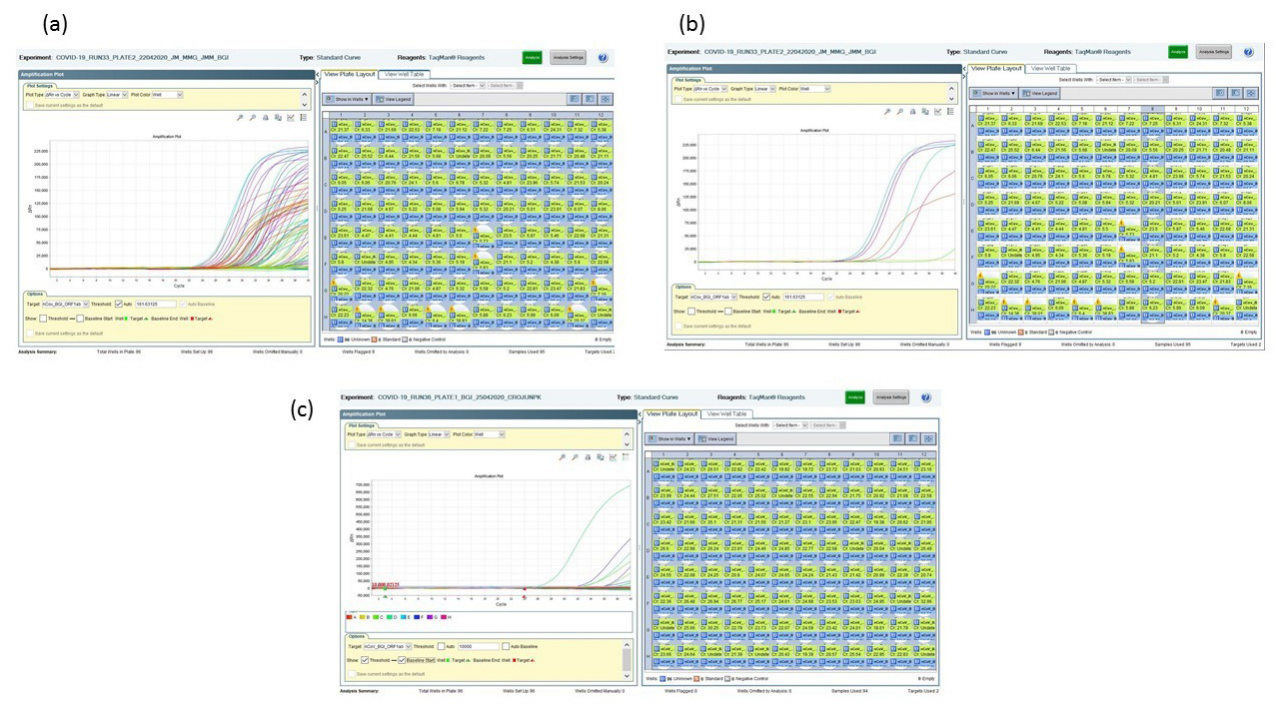
BGI assay performance. ( **A**) Multicoloured amplification curves at 0.5X of the manufacturer’s recommended volume indicating a majority positive results from a 96-sample test run. ( **B**) Selection of a single column highlighting 6 out of 8 samples were positive for SARS-CoV-2. ( **C**) Re-run of the same samples from B with 0.375X of the manufacturer’s recommended volume and 0.25 µl of the enzyme mix only detected 4 SARS-CoV-2 positive samples at a Ct value cut-off of 34.

The BGI kit produced inconsistent detection results between COVID-19 patients’ sample batches (data not shown) when we used 0.5X of the recommended reaction mix. Over 70% of the samples tested were positive ( Figures 2A and 2B), leading to a suspicion of false positive amplifications or likely contamination. A confirmatory test with Berlin E and N genes assay did not yield the equivalent number of positives. The titration of the reaction to 0.375X and scaling down the enzyme mix to 0.33X (0.5 µl) improved the specificity of the test ( Figure 2C), where there was a reduction in the number of false positives. The 0.25X titration yielded a difference of greater than two Ct values between it and the other titrations for the detected positive control RNA dilution series ( Figure 1G). In addition, the positive samples and internal control were not detected for this titration volume ( *Underlying data:* Data file 7 ^16^). The results across the three enzyme mix titrations – 0.53X (0.8µl), 0.33X (0.5µl) and 0.16X (0.25 µl) only indicated consistent detection of the dilution series of the positive control, samples and internal controls in the last two titrations ( *Underlying data:* Data file 8 ^16^; Figure 1H). Consequently, we settled for the Mix 2 combination ( Table 4), where the reaction mix was at 0.375X and the enzyme volume was at 0.16X, yielding 132 tests per kit.

### Intra-gene assay performance

The positive control RNA dilution series were used to assess the efficiency of the assays in detecting the same gene targets. We settled for the 0.375X titration to compare the performance of the assays.

**Figure 3.**
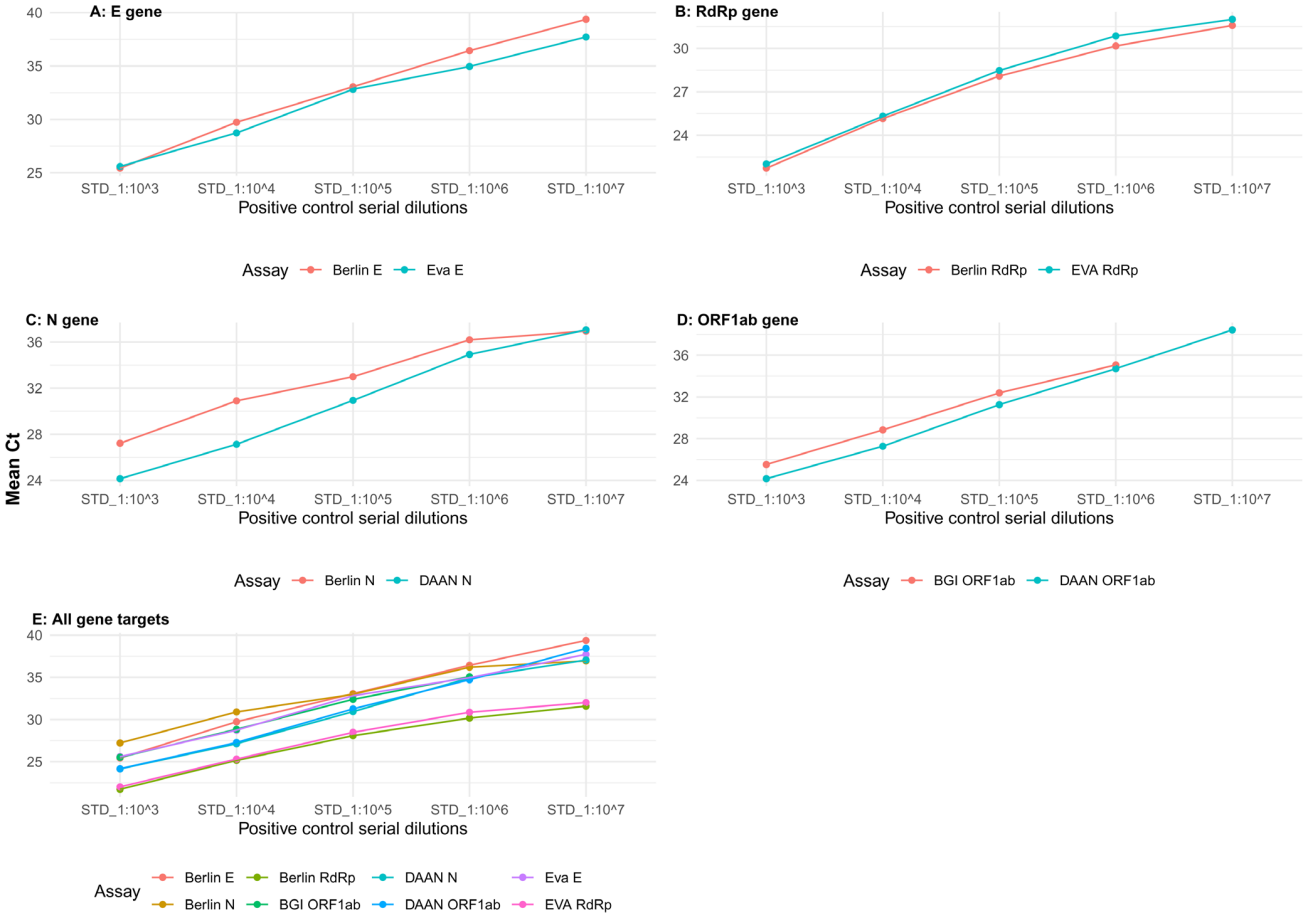
Assays concordance comparison within the same gene targets at 0.375X titration. Panels **A** and **B** show agreement in detection of E and RdRp genes respectively across all the dilution series whereas **C** and **D** show the detection of N and ORF1ab genes assays, respectively. Panel **E** highlights comparison of overall assays’ concordance across all gene targets for the same 10-fold dilution series of the positive control RNA.

The Berlin and EVAg E gene assays reported mean Ct values within range of each other and with less than 5% difference ( Figure 3A). The Berlin and EVAg RdRp gene assays showed a similar trend ( Figure 3B) with even a lower percentage difference between the mean Ct values being reported (<3%). The N gene assays had up to 13% difference in mean Ct values reported for the dilution series of the positive control. However, the DAAN N gene assay appeared to be more sensitive in detecting the virus since it had lower Ct values than the Berlin N gene assay ( Figure 3C). The DAAN ORF1ab assay also had better sensitivity than the BGI ORF1ab assay ( Figure 3D), although the mean Ct values had a maximum difference of 8.1%. Overall, there were Ct variations across these assays for the serially diluted positive controls ( Figure 3E). The RdRp gene assays appeared to have lower Ct values than the rest of the assays.

## Discussion

Our experience from performing over 15,500 tests with limited resources has allowed us to develop a series of adjustments to the primer-probe sets (Charité Berlin and EVAg) and commercial kits (BGI and DAAN) to optimize their use in SARS-CoV-2 testing. This study reports the performance of these assays following modifications on the recommended reaction volumes. Our findings suggest that the reduction in the manufacturers’ recommended volumes still allowed for detection of the virus. The 0.375X titration was the optimal volume for all the primers and probes for the gene-specific assays, and therefore recommended for resource-limited settings. The exception was the Berlin N gene assay which worked optimally at 0.5X. 

The Berlin RdRp assay generated low slope amplification curves that were characteristic of low-specificity primers. The BGI kit was the only assay whose enzyme volumes were adjusted to maximise the number of samples processed and mitigate the occurrence of false positives. The sensitivity of this assay improved when lower enzyme volumes were used as described above even though a recent publication indicated the impeccable sensitivity of the BGI kit when used according to the recommended volumes ^17^. However, according to Public Health England, the false positives in this kit could have been attributed to batch issues linked to different lot numbers ^18^. We determined that this assay was more reliable when paired with a confirmatory test from another gene target assay. The DAAN kit was efficient in detection of SARS-CoV-2 RNA, and it had the advantage over the other assays – the dual-gene target for the virus and a human gene internal control that evaluated the integrity of the sample tested and the reliability of the PCR results.

The E gene assays proved more reliable and consistent in detecting true positives. Generally, when comparing the intra-gene assay performance, we expected a variation in Ct values owing to primer design, priming efficiency and master mix differences (salt and pH).

The limitation of this study is the small sample size. These tests were conducted in the early days of the epidemic in Kenya when we had a limited supply of PCR testing kits versus a high number of samples to be tested. Consequently, we leveraged on what was available to determine the optimal parameters in our setting. We acknowledge that some of the findings cannot be generalized, nevertheless, the findings from this study enabled us to maximise the use of the limited kits and reagents available while still maintaining assay performance.

## Conclusions

We achieved approximately a 2.6-fold and 4-fold increase in the number of tests per kit for the commercial premixed kits and primer-probe sets, respectively, by adjusting the manufacturer’s recommendations on volumes following careful optimization in our laboratory. This enabled us to continuously conduct and support testing in the Coastal region of Kenya and address the challenge of inconsistencies in the supply of testing reagents. We highlight the challenges encountered in the use of the early batches of the BGI kit that we noted was prone to false positives, but this was mitigated by diluting the reagent volumes and by including an additional confirmatory assay. Due to the nature of the qPCR assay, any kit may lead to false positives and thus in addition to negative controls, a dilution series of the positive controls, a confirmatory test and a set threshold must all be included to report a positive test result more confidently. Assays should be repeated where the Ct value falls in the indeterminate range.

## Data availability statement

### Underlying data

Harvard Dataverse: An Optimization of four SARS-CoV-2 qRT-PCR assays in a Kenyan laboratory to support the national COVID-19 rapid response teams, https://doi.org/10.7910/DVN/WPZHQR ^16^.

This project contains the following underlying data:

- Data file 1 – Berlin E (FastVirus)
- Data file 2 – Berlin N (FastVirus)
- Data file 3 – EVA-g E P&P mix (FastVirus 2)
- Data file 4 – EVA-g RdRp (FastVirus
- Data file 5 – DAAN N
- Data file 6 – DAAN ORF1ab
- Data file 7 – BGI ORF1ab
- Data file 8 – BGI ORF1ab Enzyme_alterations
- Data file 9 - Berlin RdRp (Fast Virus)

Data are available under the terms of the Creative Commons Zero "No rights reserved" data waiver (CC0 1.0 Public domain dedication).
