# Supplementary material for: An optimization of four SARS-CoV-2 qRT-PCR assays in a Kenyan laboratory to support the national COVID-19 rapid response teams
Source: Wellcome Open Res. 2022 Mar 4;5:162. Originally published 2020 Jul 7. [Version 2] doi: 10.12688/wellcomeopenres.16063.2 (PMC8921690; doi:10.12688/wellcomeopenres.16063.2)
Supplement: Peer review history

# Peer review: Reviewer response for version 2

**DOI:** 10.21956/wellcomeopenres.19639.r49018

**Published:** 2022-03-14

**Authors:** Davis Nwakanma

**Affiliations:** 1 Medical Research Council Unit, The Gambia, Banjul, The Gambia; 2 London School of Hygiene & Tropical Medicine, London, UK

The response by the authors is adequate and the manuscript is substantially improved. I have no further comments.

Is the rationale for developing the new method (or application) clearly explained?

Yes

Is the description of the method technically sound?

Partly

Are the conclusions about the method and its performance adequately supported by the findings presented in the article?

Partly

If any results are presented, are all the source data underlying the results available to ensure full reproducibility?

Partly

Are sufficient details provided to allow replication of the method development and its use by others?

Yes

Reviewer Expertise:

Molecular Genetics.

I confirm that I have read this submission and believe that I have an appropriate level of expertise to confirm that it is of an acceptable scientific standard.

# Peer review: Reviewer response for version 1

**DOI:** 10.21956/wellcomeopenres.17620.r43607

**Published:** 2021-05-12

**Authors:** John Waitumbi, Esther Omuseni

**Affiliations:** 1 United States Army Medical Research Directorate-Africa/Kenya Medical Research Institute, Kisumu, Kenya; 2 Basic Science Laboratory, United States Army Medical Research Directorate-Africa/Kenya Medical Research Institute, Kisumu, Kenya

**General comments:**

- The paper has practical utility in addressing limited resources in order to sustain Covid-19 testing amidst global reagent shortages.

**Abstract:**

- **Background:** The authors are probably not aware of a paper by Altamimi *et al.* that compared the performance of 17 SARS-CoV-2 kits that included BGI and DAAN. ^1^
- **Results: **Consider rewarding the last sentence in the result’s section. For example, replace the sentence with …..“while BGI kit improved performance on dilution. This kit test results were inconsistent between batches using manufacturer's recommended volumes”.
- **Conclusion: **Consider rewording the last sentence of the conclusion to include reason/s why BGI was the best suited for comparison. Why not DAAN? Is it because BGI is singleplex?

**Introduction:**

- The authors should consider citing Altamimi et al (doi:1002/jmv_26900) that compared the performance of 17 SARS-CoV-2 kits that included BGI and DAAN. In the paper, the authors evaluated the kits in the format recommended by the manufacturer. BJI and DAAN were among the best performers.

**Methods:**

- In Tables 1-5, shouldn’t the QuantiFast multiplex RT-PCR Master mix not be included as highlighted?
**Component** **Volume**
  Nuclease-free water                                                                  4.5
  4x TaqMan® Fast Virus 1-Step Master Mix/ QuantiFast          2.5
Multiplex RT-PCR +R Kit Master Mix

 

- The paper is silent on the performance of the recommended manufacturer’s volumes. On page 6 (highlighted section), neat is indicated as 50%. Author’s should consider including data on 100%, which is the manufacturer’s recommended volume.

**Discussion:**

- The authors should consider discussion data by Altamimi *et al.* in the light of the poor performance of the BGI kit. ^1^ In the paper, BGI and DAAN were among the best performers.

Annotated PDF

Is the rationale for developing the new method (or application) clearly explained?

Yes

Is the description of the method technically sound?

Yes

Are the conclusions about the method and its performance adequately supported by the findings presented in the article?

Yes

If any results are presented, are all the source data underlying the results available to ensure full reproducibility?

Yes

Are sufficient details provided to allow replication of the method development and its use by others?

Partly

Reviewer Expertise:

Molecular epidemiology

We confirm that we have read this submission and believe that we have an appropriate level of expertise to confirm that it is of an acceptable scientific standard.

## References

[1] : Assessment of 12 qualitative RT-PCR commercial kits for the detection of SARS-CoV-2. *J Med Virol* .2021;93(5) : 10.1002/jmv.26900 3219-3226 10.1002/jmv.26900 33629747PMC8014669

# Response

**Published:** 2022-02-24

**Authors:** Khadija Said

**Affiliations:** KEMRI-Wellcome Trust Research Programme, Kenya

Thank you for your review and comments. Please find appended the responses.

**Abstract**

- Background - This manuscript was first published on 7 ^th^ July 2020 and the referenced paper was not out then ( Altamimi *et al*. 2021/).
- Results - This has been done. The ‘Results’ section has been restructured to accommodate the necessary changes.
- Conclusion - This section was restructured to convey this. We gave our findings and recommendations based on optimal volumes.

**Introduction**

- Our manuscript was first published on 7 ^th^ July 2020 and the referenced paper was not out then ( Altamimi *et al*. 2021/). The timeline difference would not fit the narrative. However, we have now referenced this paper in the ‘Discussion’ section as advised.

**Methods**

- We restructured the methods section to ensure we have comparable data for all the reagents used. Unfortunately, the QuantiFast Multiplex RT-PCR +R Kit Master Mix was only used in a limited set of assays. Consequently, we excluded these results to eliminate the confusion and misrepresentation.
- The upscaling of testing in the Coastal counties and the country at large coupled with the crippled supply chain due to the COVID pandemic meant rapid depletion of kits faster than we could replenish our stocks. This prompted us to optimize the kits in a bid to maximize the number of tests we could perform while still maintaining the integrity of the assay. As a result, we did not test the recommended manufacturer’s volumes during this optimization.

**Discussion**

- As previously mentioned, this manuscript was first published on 7 ^th^ July 2020 and the referenced paper was not out then. However, we have mentioned this in the latest version (L218-220 with hidden tracked changes). It is possible that the kit was improved by the manufacturer considering it was released around 2020. We have documented that this kit’s performance improved after the modifications were made. We believe that batch-to-batch variation based on the lot numbers would have resulted in our findings. This has been supported by an assessment done on the kit by Public Health England (see new citation 18 in the bibliography).

# Peer review: Reviewer response for version 1

**DOI:** 10.21956/wellcomeopenres.17620.r39453

**Published:** 2020-08-11

**Authors:** Davis Nwakanma

**Affiliations:** 1 Medical Research Council Unit, The Gambia, Banjul, The Gambia; 2 London School of Hygiene & Tropical Medicine, London, UK

The manuscript describes the optimisation of real-time quantitative reverse transcription PCR (qRT-PCR) protocols for SARS-CoV-2 detection with objective of increasing the reactions that could be conducted per kit (or primer-probe set) over that of the manufacturer’s recommended protocols. The authors used a set of positive and negative patient samples (four in total) and a 10-fold dilution series (up to 10 ^7^) of extracted virus culture to evaluate assay performance. On the whole two primer/probe sets and two premixed kits were evaluated.

 The rationale for this type of assay optimisation is obvious given the widespread shortage of COVID-19 diagnostic reagents across the world because of disruptions in the supply chain.

 However, I have a number of general and specific recommendation to improve the manuscript.

 In its present form, the optimisation steps are not reported in a clear and sequential format for easy comprehension. Although the processes may have been conducted in disparate steps, it would improve the clarity of the manuscript if the reporting is structured around common themes. An example could be: (1) optimisation to establish lowest concentration of reagents that produce consistent diagnostic performance (usually measured by comparison of the sensitivity and specificity against a reference, which in this case, could be the manufacturers recommended protocol; (2) determination of the combination of gene targets for optimal scoring and confirmation of positive samples; and (3) workflow optimisation to increase assay throughput.

 The use of non-standard terminologies to describe the dilutions results in unnecessary complexity for example “we tested three titration points across the four RT-PCR assays as follows: neat (50% of the manufacturer’s recommendations), 75% of the neat and 50% of the neat”. This should be simplified by designating the reagent concentration in the standard protocol as either 1X or 100%. Then the subsequent dilutions investigated would fractions of the standard and designated as .75X, .5X, .25X as the case may be.

 In reviewing the supporting files, some evaluations were conducted with a dilutions series extending only up to 10 ^4^ and others to 10 ^7^. It is difficult to understand how was the cut off for the Berlin RdRP assay was determined to be 31, given that the limit of detection (LOD) was not reached in the evaluation shown in data files 5 & 6 where the lowest dilution of 10 ^4 ^was detected with a CT of 26-27.

 Figure 1 is particularly difficult to follow given the amount of information contained. This is not helped by ignoring the chronological order in the explanatory text which begins with graph ‘D’ instead of ‘A’.

 A large part of the discussion section is devoted to reporting optimisation steps (which would fit better under methods) instead of highlighting the implications and potential impact of these changes, for example, “For quality control during testing, we included in every assay a negative template control, two positive extraction controls (a neat and 1 in 10 dilution) and 2 negative controls placed randomly (but not near each other) across the wells of a 96-well format plate”.

 Overall, the manuscript will benefit from reporting the optimisation steps in a structured format, simplifying the ‘nomenclature’ used in describing the dilutions, reviewing the discussions to remove description of the methods, explaining the rationale for the cut-off CTs for the various assays (gene targets) in the absence of determining the LOD and by simplifying Figure 1.

Is the rationale for developing the new method (or application) clearly explained?

Yes

Is the description of the method technically sound?

Partly

Are the conclusions about the method and its performance adequately supported by the findings presented in the article?

Partly

If any results are presented, are all the source data underlying the results available to ensure full reproducibility?

Partly

Are sufficient details provided to allow replication of the method development and its use by others?

Yes

Reviewer Expertise:

Molecular Genetics.

I confirm that I have read this submission and believe that I have an appropriate level of expertise to confirm that it is of an acceptable scientific standard, however I have significant reservations, as outlined above.

# Response

**Published:** 2022-02-24

**Authors:** Khadija Said

**Affiliations:** KEMRI-Wellcome Trust Research Programme, Kenya

Thank you for your review and comments. Please find appended the responses.

 a) Response to comment 1: The manuscript has been restructured and centered around establishing the lowest volumes of the reagents required to generate consistent diagnostic performance while increasing assay throughput. For a clear narrative, the four assays have been introduced in the ‘Introduction’ section (L25-32 in the hidden tracked version of the manuscript). The optimization method has been initially defined in the ‘Real-time PCR assay modifications’ section (L57-60) within the ‘Methods’ section. The adjustments that make up our optimization steps have been explained in two sections based on the type of assay - primer-probe sets (L63-71) and commercial kits (L82-96).

 b) Response to comment 2: Noted and modified as suggested. Herein, we refer to the manufacturer-recommended volumes at 1X, and our titration points are referred to by 0.5X, 0.375X and 0.25X to represent 50%. 37.5% and 25% of the manufacturer-recommended volumes respectively.

 c) Response to comment 3: Included the relevant data for Berlin RdRp comprising all the dilution series tested up to 10 ^7^. Refer to Updated Data File 9. However, due to issues with Berlin RdRp curves we excluded the Ct cut-off of this assay in Table 6.

 d) Response to comment 4: The plot in Figure 1 was replaced by a more succinct one. Ensured the chronological order of the accompanying plots and texts was maintained.  

 e) Response to comment 5: For this part of the discussion section, the idea was to report our experience and emphasize on the importance of including controls in the qRT-PCR assays especially during optimization. However, following the restructuring of the ‘Discussion’ section this was excluded and the message adheres to the context.
